# Seasonal changes characterise the shark and ray assemblages in a subtropical shallow sandy habitat in the iSimangaliso Wetland Park, South Africa

**DOI:** 10.7717/peerj.15636

**Published:** 2023-07-14

**Authors:** Jessica A. Ferreira, Julie A. Alberts, Grant Smith, Anthony T.F. Bernard, Mário J. Pereira, Lauren De Vos

**Affiliations:** 1 Departamento de Biologia, Universidade de Aveiro, Aveiro, Portugal; 2 Unaffiliated, Tampa, FL, United States of America; 3 Sharklife Conservation Group, Sodwana Bay, KwaZulu-Natal, South Africa; 4 Department of Zoology and Entomology, Rhodes University, Makhanda, Eastern Cape, South Africa; 5 SAIAB (South African Institute for Aquatic Biodiversity), Rhodes University, Makhanda, Eastern Cape, South Africa; 6 Departamento de Biologia & CESAM (Centre for Environmental and Marine Studies), Universidade de Aveiro, Aveiro, Portugal; 7 Save Our Seas Foundation, Geneva, Switzerland

**Keywords:** Elasmobranchs, Species diversity, Relative abundance, Sandy habitat, Environmental drivers, Seasonality, Stereo-BRUVs

## Abstract

Understanding how environmental drivers influence shark and ray spatial and temporal patterns can provide crucial knowledge for their evidence-based protection and long-term monitoring. However, information on which drivers of variation are most important for elasmobranch communities on soft sediments is limited. Using baited remote underwater stereo-video systems (stereo-BRUVs), we investigated how seasonal and environmental variables affected the elasmobranchs of the iSimangaliso Wetland Park marine protected area (MPA) in South Africa (SA). In total, 11 species were identified from 48 sites between 12 m and 33 m water depth in a sandy habitat. While species richness was similar across seasons, the total abundance of elasmobranchs recorded was higher in winter than summer. The species assemblage composition varied significantly between seasons, with the Human’s whaler shark *Carcharhinus humani* prevalent in summer and the Critically Endangered whitespotted wedgefish *Rhynchobatus djiddensis* more abundant during winter. Most species were sighted throughout the entire depth range, but rays were more common in shallower waters (< 25 m depth), while *C. humani* and *R. djiddensis* were more common in the deeper depth zone of this study. This research provides baseline information about this previously unexplored sandy habitat for elasmobranchs in a site of regional and global significance. Records of species of conservation concern in the sampling area highlight the importance of protecting sand environments within an MPA.

## Introduction

The impacts of changing seasons in the marine environment can be perceived at several scales, from local ecosystems (Scrosati & Ellrich, 2020) to entire oceans (Lee & Jin, 2021; Liao et al., 2022). In turn, environmental variations can impact different trophic levels, from microorganisms (Kudryavtseva et al., 2019) to megafauna (Rayment, Dawson & Slooten, 2010), including elasmobranchs (Blaison et al., 2015; Ketchum et al., 2014).

A change in temperature in the ocean is one of the most obvious and predictable seasonal variations (Jo et al., 2022). Since most sharks and rays are ectotherms (rely on the environment to determine their body temperature), environmental temperature variations can be a key driver for their physiology (Bernal et al., 2018; Silva-Garay & Lowe, 2021) and spatial/temporal distribution (Carlisle & Starr, 2009; Elston et al., 2022; Schlaff, Heupel & Simpfendorfer, 2014). For example, De Vos et al. (2015) found temperate shark diversity to be higher in summer than winter, and Brooks et al. (2013) encountered the same seasonal pattern for the abundance of Caribbean reef sharks (*Carcharhinus perezi*). Elasmobranchs also respond to biological cues to exploit seasonally abundant food sources (Hutchings et al., 2010; Dudley & Cliff, 2010), and mate/pup in specific locations and times of the year (Jirik & Lowe, 2012; Dicken, 2006; Olbers & Cliff, 2017). Accordingly, seasonality reveals itself as a proxy for a host of environmental and biological cues, which makes it an influencing factor of changes or patterns in elasmobranch communities.

However, not all sharks and rays are expected to move in response to seasons. Environmental variables such as depth, water quality and habitat availability also drive the community structure and species diversity of elasmobranch assemblages (Heupel et al., 2014). Nevertheless, rays and benthic sharks are most likely to be present year-round (Tilley & Strindberg, 2013), while the populations of more mobile elasmobranchs can fluctuate throughout time (Taylor, Sumpton & Ham, 2011; Ruiz-Abierno et al., 2021). Alternatively, some species exhibit site fidelity by remaining in the same region all year, but their distribution within the area can change (Hammerschlag et al., 2012; Vaudo & Heithaus, 2012); for instance, between shallower and deeper zones (Ketchum et al., 2014; Le Port, Lavery & Montgomery, 2012; Vianna et al., 2014). Studies have shown that the species richness and diversity of shark assemblages change with depth (Lucifora et al., 2012), and that larger apex shark species, such as tiger sharks (*Galeocerdo cuvier*), are more common in deeper waters (Lester et al., 2022). Additionally, this species and sandbar sharks (*Carcharhinus plumbeus*) demonstrate ontogenetic changes in depth distributions (Afonso & Hazin, 2015; McAuley et al., 2007). Understanding how environmental drivers affect sharks and rays is important in the context of their conservation (Peterson & Grubbs, 2020; Schlaff, Heupel & Simpfendorfer, 2014), especially to inform species-specific regulations.

Several studies suggest that well-enforced MPAs effectively protect elasmobranch populations (Ferretti et al., 2008; Heupel et al., 2014; MacNeil et al., 2020; Micheli et al., 2012), and even present 14 times more shark biomass than fished areas (Edgar et al., 2014). The management of elasmobranchs is increasingly being incorporated into the planning of MPAs (Bergmann et al., 2022; MacKeracher, Diedrich & Simpfendorfer, 2019), and the positive effect of no-take zones on endangered shark and ray species diversity and abundance is clear (Albano et al., 2021; Da Silva et al., 2013). The long-term monitoring of biodiversity and key species groups is important to demonstrate the effectiveness of MPAs and to guide adaptive management.

In the iSimangaliso Wetland Park (iSimangaliso) and World Heritage Site MPA, on the east coast of SA, sharks and rays remain a significantly under-studied group, with few elasmobranch studies undertaken in this region (Cliff et al., 2007; Daly et al., 2021; Dicken, 2014; Gifford et al., 2007; Olbers & Cliff, 2017). While coastal coral reef ecosystems are the focus (Floros, Samways & Armstrong, 2004; Floros, Schleyer & Maggs, 2013; Schleyer et al., 2018), there is a lack of similar attention on soft sediment areas, which can be valuable to elasmobranchs, particularly to bottom-dwellers (Pennino et al., 2013; Pierce, Scott-Holland & Bennett, 2011; Vaudo & Heithaus, 2009; Vaudo & Heithaus, 2012). For instance, sandy habitats provide sharks with foraging and reproduction areas, and offer juvenile rays important nursery areas (Martins et al., 2020; Parton et al., 2023). Thus, understanding the importance of different habitats for shark and ray species can inform conservation management strategies (Knip, Heupel & Simpfendorfer, 2012; White et al., 2015).

This study consisted of seasonal surveys (winter of 2021 and summer of 2022) of elasmobranch communities in a shallow sandy area within the iSimangaliso MPA. The main objectives were to assess shark and ray assemblages, and to evaluate the influence of season, depth, temperature, and current strength on species composition. To achieve this, species richness and relative abundance of elasmobranchs were assessed using baited remote underwater stereo-video systems (stereo-BRUVs).

## Materials & Methods

### Study area

This research was conducted in the iSimangaliso Wetland Park, an MPA on the north-eastern coast of SA, in KwaZulu-Natal (KZN), in the Western Indian Ocean (WIO). iSimangaliso is a large and transboundary MPA encompassing different protection levels, each permitting varying degrees of human activity. For logistical purposes, a small subsection of the MPA was sampled in this study. The study area is approximately 15 km in length and 2 km wide, and is a shallow zone with a northern limit at Jesser Point, a boat launch site in Sodwana Bay. It is located in the iSimangaliso Offshore Controlled Pelagic Linefish Zone North (IOCPLZN) management zone, in which diving and pelagic game fishing are allowed, but anchoring, bottom-fishing, night fishing and fishing for elasmobranchs are forbidden.

Unlike the surrounding zones where the substrate type is mainly shallow coral reefs, this area’s seafloor is primarily sand and has a submarine canyon in the middle of its length known as the Diepgat canyon. Although the study area has never been researched, photic soft sediments of KZN are known to be dominated by macrobenthic invertebrates (such as crabs and echinoderms) and also Sciaenidae and Haemulidae fish species (MacKay & Untiedt, 2014; Mann & Fennessy, 2014). In turn, Diepgat and other canyons of this region have revealed a great species richness of fish, including rays and coelacanths (Geldenhuys, 2015; Roberts et al., 2006).

This research was fully approved by the Department of Environment, Forestry and Fisheries of the Republic of South Africa (permit for the purposes of a scientific investigation or practical experiment in terms of section 83 of the Marine Living Resources Act, 1998 (act no. 18 of 1998), Res2021-25 and Res2022-60), and by the iSimangaliso Wetland Park Authority (Research Agreement with Sharklife Conservation Group and with Wildlands Conservation Trust (WILDTRUST) for the Oceans Alive project). This research was used for a Master’s thesis (Ferreira, 2022).

### Baited remote underwater stereo-video system (stereo-BRUVs) surveys

**Figure 1:**
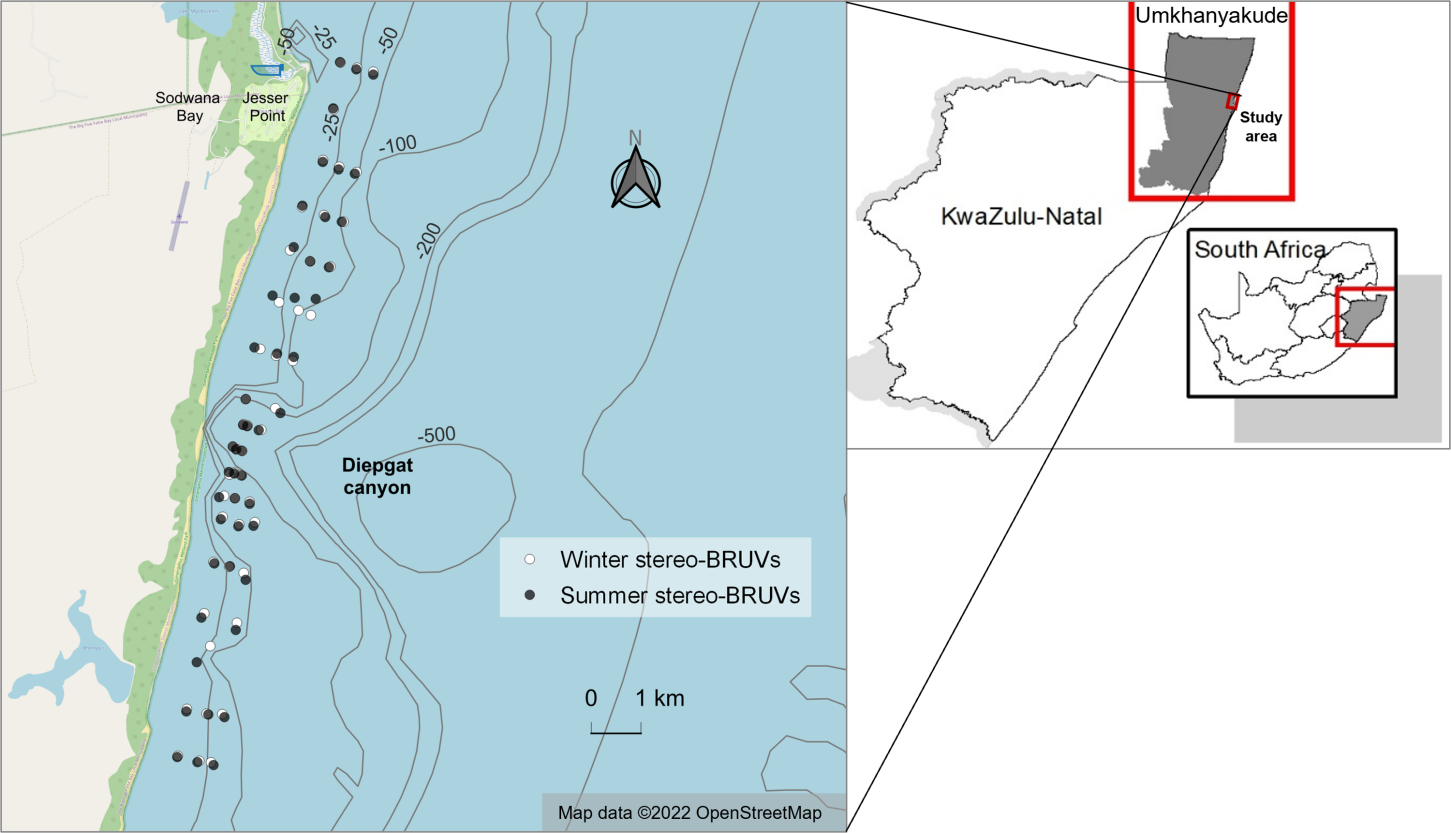
Winter and summer baited remote underwater stereo-video systems (stereo-BRUVs) sampling sites. The overlap between winter (white) and summer (black) sites is due to their similar geographic coordinates. Map data from OpenStreetMap (2022). Bathymetry data from  GEBCO (2021). Borders from Municipal Demarcation Board (2018).

Surveys using stereo-BRUVs were performed during winter (July–September 2021) and summer (January–March 2022). To guarantee similar sampling efforts throughout the photic zone, the sampling sites were selected following depth contour lines while driving the boat from shallower to deeper areas. These sites were later divided into three categories: “shallow”, between 12 and 20 m; “mid-depth” between 20.1 and 25 m; and “deep”, between 25.1 and 33 m.^1^ 1Depth contour lines in Fig. 1 are visual representations and continuously change with tides and seasons, with the only certain depth being the one taken at the moment of sampling.This sampling approach was the same for both seasons, as comparable locations were sampled in winter and summer. To ensure that the comparisons across seasons were consistent, only samples of equivalent depths were included in the final analysis. Consequently, 48 stereo-BRUVs from each season were used for analysis (Fig. 1). Within these 48 sites for summer and winter, 19 sites were in the north zone, 17 were located near the submarine canyon (distance less than 1.8 km), and 12 were in the south zone of the study area.

The deployments performed during the same day (up to six deployments) were located at least 350 m apart in an inshore to offshore direction to establish a sufficient minimum distance between sampling sites to prevent individual elasmobranchs from being recounted, and thereby avoiding pseudo-replication (Haggitt, Freeman & Lily, 2014; Langlois et al., 2020). In the longshore direction, each deployment was located 1 km apart from the next in order to fully sample the study area. Near the canyon, the longshore distance was reduced to 500 m to increase sampling efforts in that region.

The stereo-BRUVs were equipped with pairs of GoPro Hero 6 or 7 cameras (50 or 60 frames per second, resolution of 1080p, and linear setting). Calibration using SeaGIS CAL software (SeaGIS, 2022a) was performed before sampling to ensure the cameras’ overlapping field of view enabled stereo-measurement (Harvey & Shortis, 1995). The cameras were installed in a steel trapezoidal structure, with one 1 kg weight attached to each leg for stability on the seafloor. In front of the cameras, a 1 m length rod with a perforated PVC bait box at the end contained 1 kg of chopped sardines *Sardinops sagax*. The stereo-BRUVs were manually deployed to the seafloor through a rope with a surface buoy.

At the time of each deployment, geographic coordinates were registered by the vessel’s GPS (Hook-7 Lowrance GPS system, Lowrance Ltd, Tulsa, OK, USA), and depth (m) and sea surface temperature (SST (°C)) were taken by the inbuilt transducer. Thirty-eight winter stereo-BRUVs and 17 summer stereo-BRUVs were fitted with a temperature logger (iButton, Maxim Integrated DS1921H-F5#, Digital Temperature Sensor; Whitewater, WI, USA) to record sea bottom temperature (SBT (°C)), while 41 winter stereo-BRUVs and 38 summer stereo-BRUVs had current strength (km/h) registered by a current meter (Garmin Colorado 300 GPS, Olathe, KS, USA). The standard soak time was one hour minimum (Langlois et al., 2020), which allowed for multiple deployments across the study area on a day.

### Video analysis

All recorded videos were analysed until the 60 minutes of footage were completed. During that time, most elasmobranchs were identified to species level (using Ebert, Dando & Fowler, 2021; Last et al., 2016) but some could only be identified to family level. The latter records were omitted from the data analysis. It was not possible to assess to species level some rays of the *Himantura uarnak-leoparda* species complex with honeycomb/spotted patterning. As such, these were identified as *Himantura* spp. and were included in the data analysis. To avoid double counting the same individual, MaxN was registered for each species, for each stereo-BRUVs sample. The MaxN measure is the maximum number of individuals of a species seen during the 60-minute sample in the same video frame (Cappo et al., 2003). The sum of all MaxN for each species divided by the total number of samples determined relative abundance per species. Species diversity was considered species richness (number of species). Underwater visibility (cm) was considered the furthest visible distance possible that still allowed for an animal to be identified, and it was measured through SeaGIS EventMeasure 5.71 software (SeaGIS, 2022b).

### Statistical analysis

Kruskal-Wallis tests and Dunn’s multiple comparisons tests (uncorrected Dunn’s test for comparisons between winter and summer SBT and within summer SBT, given that the corrected *p*-values did not allow to assess the significant differences) were used to analyse environmental variables and assess significant differences according to season, location and depth (*p* < 0.05 for significant differences). Unpaired Mann–Whitney U tests were adopted to check for significant seasonal differences in underwater visibility, and also in the number of elasmobranchs sighted per deployment. These analyses were performed in GraphPad Prism 9 (GraphPad, 2022).

To determine the effect of season and environmental variables in elasmobranch composition, multivariate statistics were performed. Biological data were log(x+1) transformed to ensure the trend was still detectable in spite of dominant species. Missing values in SBT and current strength data were replaced with the correspondent seasonal mean value. A canonical correspondence analysis (CCA) was used to visualise the relationship between the elasmobranch assemblages and SBT, depth and current strength (SST was excluded since SBT reflected the exact temperature of the species sightings). All explanatory variables were included, but summarizing effects allowed to identify which ones contributed significantly to species composition (*p* < 0.05). The CCA was preceded by a detrended correspondence analysis (DCA) to assess the main gradients along the species matrix (not including environmental variables). The results of the DCA suggested preference for a CCA (a unimodal ordination method) instead of a redundancy analysis (RDA) (a linear ordination method) (Zelený, 2022). These two analyses were run in Canoco 5.1 (Ter Braak & Smilauer, 2012).

A Bray-Curtis resemblance matrix (with a dummy variable = 1) was created among samples to obtain similarity in elasmobranch composition and abundance. Sequential permutational multivariate analyses of variance (PERMANOVAs) were run on this matrix to assess the effect of depth (shallow/mid-depth/deep), location (North/canyon/South), season (winter/summer), and the interactions of these variables in elasmobranch composition. By employing a sequential PERMANOVA, the effect of season could be tested after accounting for variation in the assemblage caused by depth and location. A PERMDISP was used to check if the levels of the factors differed in terms of the variability within each level, and pairwise tests were run for the significant factors in PERMANOVA. The results were assessed under the pseudo-F statistic with 999 random permutations (*p* < 0.05). These analyses were conducted in PERMANOVA+, an extension software in PRIMER-e 6 (Anderson, Gorley & Clarke, 2008).

One-way similarity percentages analyses (SIMPERs) were run, also in PRIMER-e 6, on the transformed biological data to identify the contribution of each species to the Bray-Curtis dissimilarity between depth zones and the two seasons (Clarke, 1993).

## Results

### Sample sites and environmental variables

Depth ranged from 12.2 to 33.0 m in winter (mean [x] ± standard deviation [SD] = 22.30 ± 6.50 m) and from 12.7 to 32.0 m in summer (x ± SD = 21.97 ± 6.54 m). A total of 21 winter and summer deployments were in the “shallow” depth category, 11 winter and summer deployments were in the “mid-depth” depth category, and 16 winter and summer deployments were deployed in the “deep” depth category.

**Figure 2:**
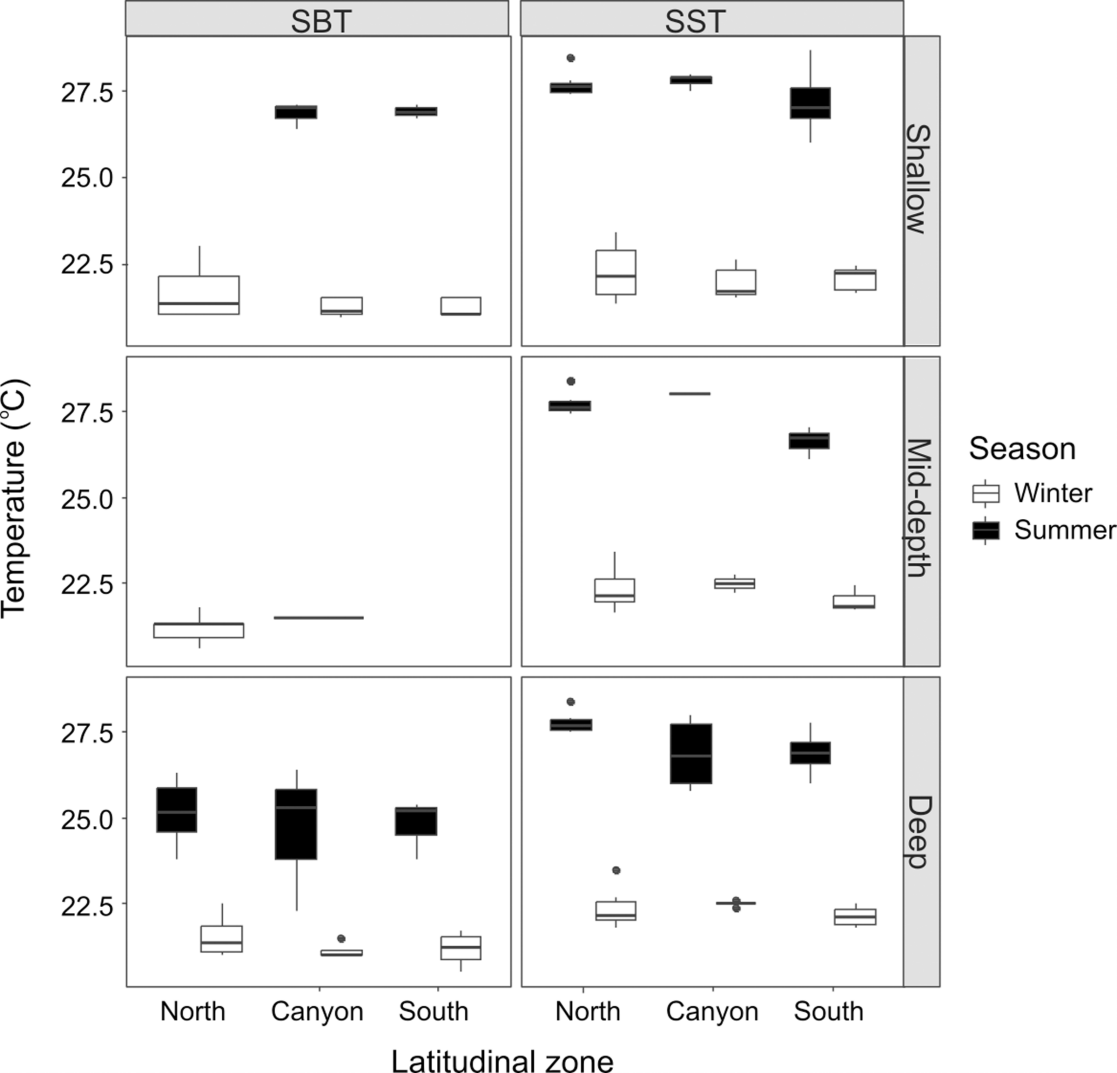
Seasonal sea bottom temperature (SBT) and sea surface temperature (SST). Winter and summer temperatures in the north, near the canyon, and south zones of the study area, within the “shallow”, “mid-depth” and “deep” depth categories.

The average SST was lower during winter (22.21 ± 0.54 °C) than summer (27.45 ± 0.70 °C), and the same pattern was verified for SBT (21.34 ± 0.52 °C for winter; 25.49 ± 1.35 °C for summer). Although there were no significant differences across locations and depth categories for winter SST (Kruskal-Wallis statistic (K-W) = 9.88; *p* = 0.273), winter SBT (SBT: K-W = 8.37, *p* = 0.301) or summer SST (K-W = 15.18, *p* = 0.056), summer SBT was significantly different (K-W = 10.04, *p* = 0.011) between the “shallow” and “deep” zones near the canyon region (*p* = 0.0288) (Fig. 2). Winter SST and SBT were significantly different (K-W = 42.89, *p* = 0.0003), but not within the same location and depth category, while summer SST and SBT were significantly different (K-W = 37.19, *p* = 0.0002) in the “shallow” zone in the North (*p* = 0.0006). Both SST (K-W = 76.66, *p* < 0.001) and SBT (K-W = 36.14, *p* = 0.0003) were significant different between winter and summer, for example in the “shallow” zone near the canyon (SST: *p* = 0.0004; SBT: *p* = 0.001). In addition, SBT also varied considerably across seasons in the “shallow” zone in the South (*p* = 0.009), as well as in the “deep” zone in the North (*p* = 0.022) and South (*p* = 0.025).

**Figure 3:**
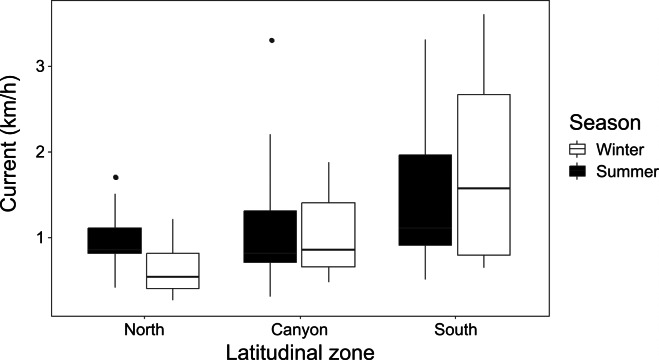
Seasonal current strength. Winter and summer current strength in the north, near the canyon, and south zones of the study area.

Current strength (1.08 ± 0.79 km/h for winter; 1.23 ± 0.73 km/h for summer) was significantly affected by location (K-W = 16.26, *p* = 0.006), with the North and South zones differing during winter (*p* = 0.015), and the North during winter differing from the South during summer (*p* = 0.022) (Fig. 3). Underwater visibility (768.1 ± 528.9 cm for winter; 684.0 ± 325.7 cm for summer) was not significantly different between seasons (Mann–Whitney *U* [M-W U] =1026, *p* = 0.675).

### Species richness and relative abundance

Approximately 77.08% of winter deployments (*n* = 37) and 66.67% of summer deployments (*n* = 32) recorded at least one sighting of an elasmobranch. The number of observed individual elasmobranchs per deployment (1.40 ± 1.12 individuals/deployment in winter; 1.23 ± 1.17 individuals/deployment in summer) was not significantly different between seasons (M-W *U* = 1042, *p* = 0.408).

**Figure 4:**
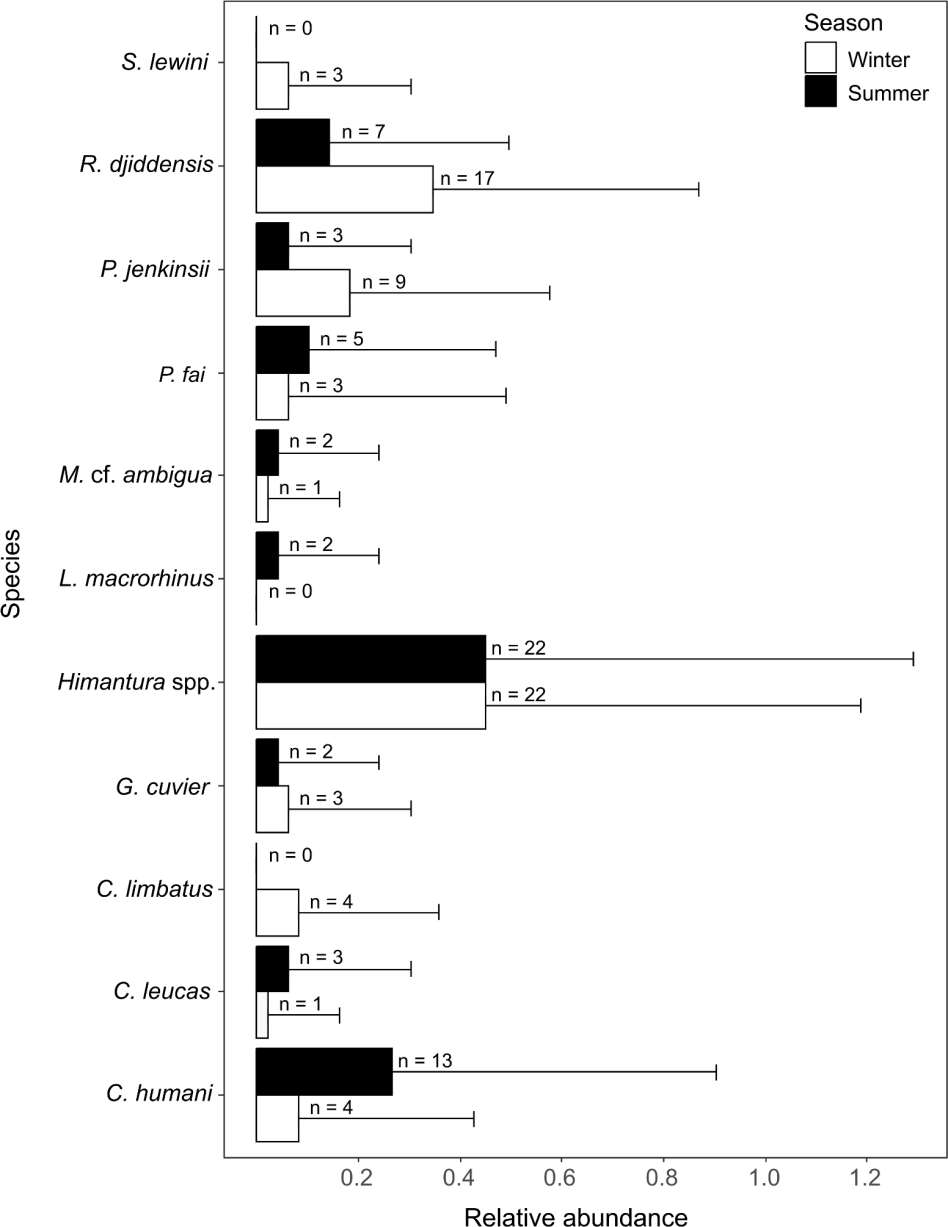
Relative abundance (±SD) of each elasmobranch species found during winter and summer. The n values in front of the bars represent the sum of MaxN of each species during each season.

A total of 10 species were identified in winter (five shark species, *n* = 15; five ray species, *n* = 52) and nine were identified in summer (four shark species, *n* = 20; five ray species, *n* = 39). All rays and three species of sharks were present in both seasons. The common blacktip shark *Carcharhinus limbatus* and the scalloped hammerhead shark *Sphyrna lewini* were only recorded in winter, and the sliteye shark *Loxodon macrorhinus* was only seen in summer (Fig. 4).

### Elasmobranch community analyses

**Figure 5:**
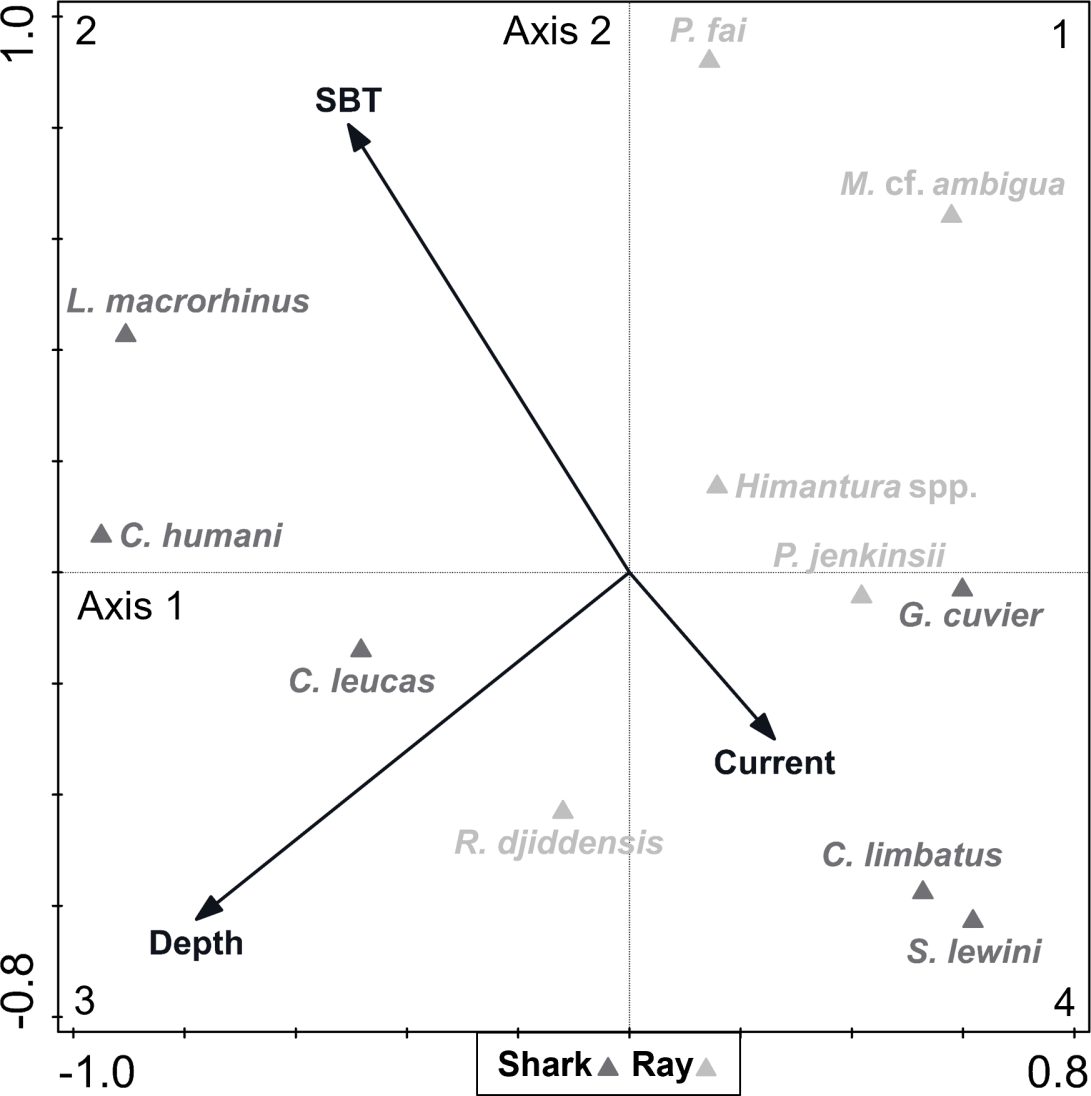
Canonical correspondence analysis (CCA) biplot representing the effect of environmental variables—current strength (current), depth and sea bottom temperature (SBT)—on species composition. Environmental variables are represented by black arrows and species are represented by dark (shark) and light (ray) grey triangles.

A DCA on the biological data revealed that species constituted a heterogenous dataset, since there was a strong ecological gradient (gradient length of axis 1 = 4.28, given that a dataset is considered heterogenous if gradient length of axis 1 > 4). The CCA analysis (eigenvalue of axis 1 = 0.261; eigenvalue of axis 2 = 0.162; explained fitted variation of axis 1 = 55.63%; explained fitted variation of axis 2 = 90.06%) revealed that the continuous environmental variables accounted for 7.35% of the variation in species composition (Fig. 5). Depth (explained 3.50% of the variation, *p* = 0.012) and SBT (explained 2.80% of the variation, *p* = 0.036) were variables that contributed significantly to elasmobranch variation, while current strength (explained 1.00% of the variation, *p* = 0.608) was not considered a significant explanatory variable to species composition. The depth vector was directed towards quadrant 3 (negative segments of axes 1 and 2), while the SBT vector varied towards quadrant 2 (negative segment of axis 1 and positive segment of axis 2), and the current strength vector varied towards quadrant 4 (positive segment of axis 1 and negative segment of axis 2).

Most species (including all rays except the whitespotted wedgefish *Rhynchobatus djiddensis*) were located between quadrants 1 and 4, which corresponded to shallower depths. On the contrary, the Human’s whaler shark *Carcharhinus humani* and *L. macrorhinus* showed up on quadrant 2, linked to deeper waters. The bull shark *Carcharhinus leucas* and *R. djiddensis* were also detached in quadrant 3, in deeper zones. In turn, the pink whipray *Pateobatis fai* and *L. macrorhinus,* and to a lesser extent *C. humani* and the sharpnose whipray *Maculabatis* cf. *ambigua*, seemed to be associated with higher SBT (summer), while *C. limbatus* and *S. lewini* were associated with lower SBT (winter).

The three-factor sequential PERMANOVA with categorical variables showed that depth and season (in this sequence) had a significant effect on elasmobranch composition (Table 1). The PERMDISP tests comparing variability within factors revealed non-significant results, which indicated that the variability in elasmobranch composition was similar among the factor levels.

**Table 1:** Permutacional analysis of variance (PERMANOVA) results testing the effect of season, depth, location, and the interactions of the three factors in elasmobranch composition.

**Model input factors**	**df**	**Pseudo-F**	***P***
Depth	2	3.165	0.005
Location	2	1.584	0.139
Season	1	3.093	0.016
Depth × Location	4	1.040	0.404
Depth × Season	2	1.261	0.293
Location × Season	2	1.694	0.100
Depth × Location × Season	4	1.254	0.262

**Notes.**

df degrees of freedom

Underlined *p*-values indicate significance at *p* < 0.05.

**Figure 6:**
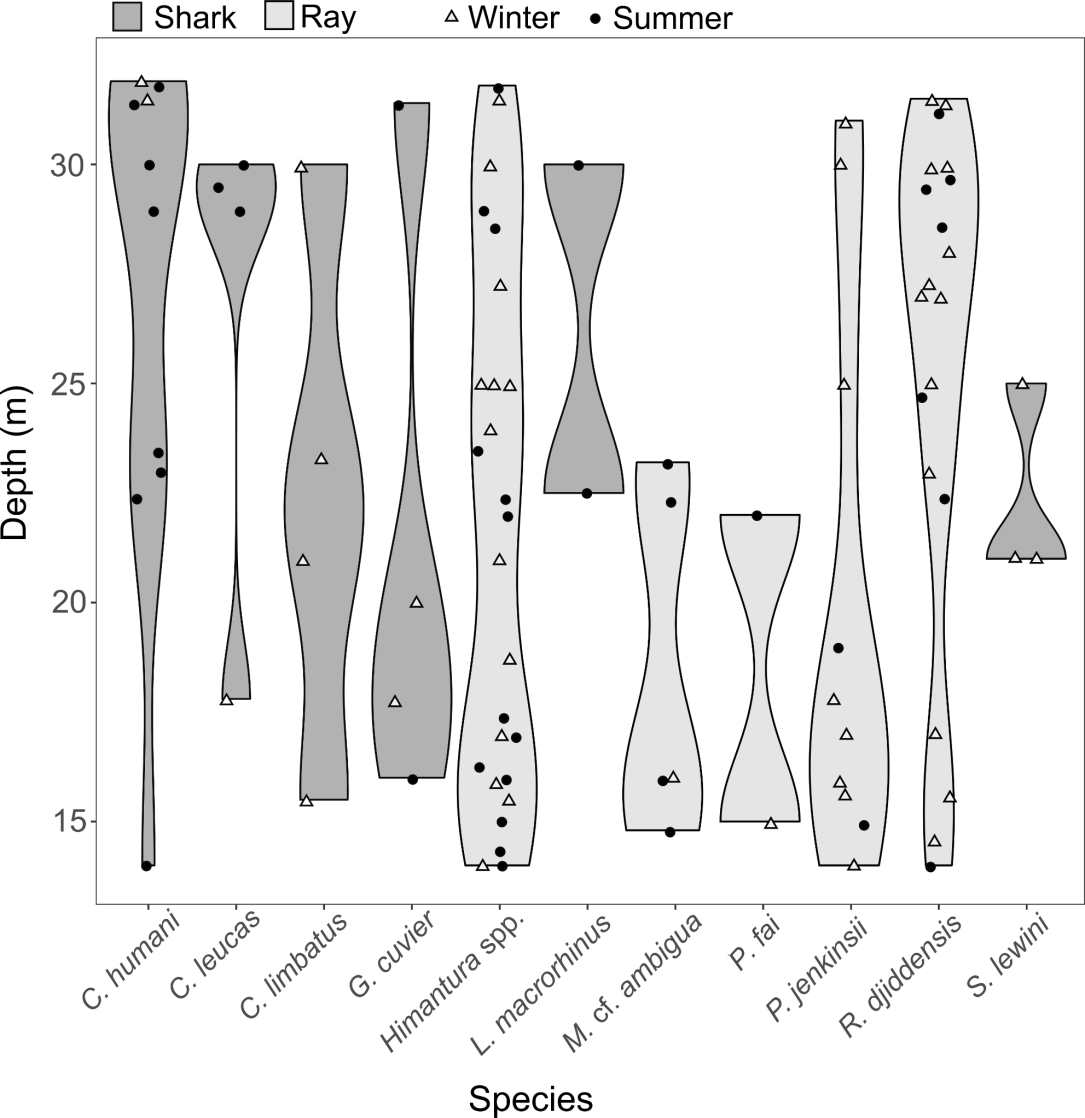
Distribution along depth of the sighted shark and ray species. The white triangles (winter) and black dots (summer) provide the depths of the samples where each species was detected.

Pairwise tests revealed that the effect of depth was driven by the difference between “shallow” and “deep” zones (*p* = 0.002), while there were no significant differences between the “mid-depth” zone and either the “shallow” (*p* = 0.279) or “deep” zone (*p* = 0.058). The SIMPER analysis revealed that the difference between the “shallow” and “deep” zones was driven by higher occurrence of *R. djiddensis* (dissimilarity x, x/SD: 24.47, 0.77) and *C. humani* (dissimilarity: 12.26, 0.54) in the “deep” zone, and higher occurrence of *Himantura* spp. (dissimilarity: 22.89, 0.81) and the Jenkin’s whipray *Pateobatis jenkinsii* (dissimilarity: 10.76, 0.51) in the “shallow” zone (Fig. 6).

The SIMPER analysis indicated that the seasonal variation in the elasmobranch assemblage was driven by higher rates of detection for *R. djiddensis* (dissimilarity: 20.04, 0.68) and *P. jenkinsii* (dissimilarity: 9.88, 0.48) in winter than summer (Fig. 4). On the other hand, *C. humani* was a characteristic species of the elasmobranch assemblage in summer (dissimilarity: 10.23, 0.50), but not winter (Fig. 4). *Himantura*. spp. was the most abundant elasmobranch recorded during the study with similar abundances in winter (x ± SD = 0.46 ± 0.74) and summer (0.46 ± 0.85).

## Discussion

### General diversity and species findings

This study documented 11 species of elasmobranchs with a similar diversity of sharks (six species) and rays (five species). Rays were more common than sharks, highlighting the importance of unconsolidated habitats for these organisms. It has been demonstrated that sandy areas can be crucial to batoid species, especially juveniles, as they seem to concentrate more in soft sediment than in coral reefs (Martin et al., 2012; Martins et al., 2020), while mature individuals are more easily found in both types of habitat (Aguiar, Valentin & Rosa, 2009).

This study found that *R. djiddensis* was a notably abundant species in the study area during winter. This wedgefish is listed as globally “Critically Endangered” and has suffered an estimated population decline of over 80% over the past 45 years (Kyne, Gledhill & Jabado, 2019). In SA, its population has been described as “Endangered”, with the main threats in KZN being fisheries—*e.g.*, shore angling—and the bather protection nets (locally known as shark nets) located south of the study area (Daly et al., 2020; Pradervand et al., 2007). Since the mature individuals of this species can move long distances, they are also vulnerable to threats across the border in Mozambique (Jordaan et al., 2021; SAAMBR, 2022). Additionally, an illegal fin trade of *R. djiddensis* and other endangered sharks in southern Africa has been recently unveiled (Asbury et al., 2021). Accordingly, the current results strengthen the fact that the iSimangaliso MPA is important to the conservation of this species (Jordaan et al., 2021). Finding endangered elasmobranchs in this region supports the prohibition of elasmobranch fishing in the study area. This level of protection is particularly relevant considering that there is no legislation that forbids shore angling in the “Controlled” zones of iSimangaliso (iSimangaliso Wetland Park Authority, 2022).

*Himantura* rays with a honeycomb/spotted pattern were the predominant elasmobranchs in the area. In this study, elasmobranch identification was based on characteristics visible in the video footage, and rays of the *H. uarnak-leoparda* species complex could not be specifically categorized under one species. In fact, the genetic relationships within this species complex—to which the reticulate whipray *Himantura uarnak* and the leopard whipray *Himantura leoparda* belong (both extant in SA)—are still to be clarified (Arlyza et al., 2013; Borsa et al., 2021). The study area provides the opportunity for taxonomic studies to elucidate on whipray identification.

*Carcharhinus humani* was the most abundant of the six shark species. The Human’s whaler shark, previously thought to be the widespread *C. sealei*, was first described in 2014 and so far, it is the only species from the *C. sealei* subgroup to inhabit the WIO (White & Weigmann, 2014). This shark is a poorly known species, which reflects in its “Data Deficient” conservation status (Pollom et al., 2019), but this study confirmed that, as with *C. sealei*, *C. humani* occurs in photic and soft sediment habitats (Dulvy et al., 2021; Pollom et al., 2019). The larger species of sharks were also not abundant in the study area, which makes sense since these species are highly mobile, with average travelling distances between 86 and 267 km, as evidenced by the Oceanographic Research Institute (ORI) tagging and recapture programme in SA (iSimangaliso Wetland Park Authority, 2022).

### Seasonality and environmental predictors of elasmobranch diversity and abundance

Sightings of sharks and rays were higher in winter than in summer. Species richness and diversity was similar across seasons, as the majority of species were common to both. While the univariate metrics were relatively stable, when species identity was considered, a significant seasonal pattern was evident with the “Critically Endangered” *R. djiddensis* being more common in winter and the relatively unknown *C. humani* being more common in summer. The remaining species showed little seasonal variation or had insufficient data to confidently identify patterns.

*Rhynchobatus djiddensis* is known to be most abundant in central and southern KZN during summer and to concentrate northwards, possibly into Mozambique, during winter (Daly et al., 2020; Jordaan et al., 2021). The current findings suggest that at least some wedgefishes remain in SA waters during winter, where they are under the protection of the iSimangaliso MPA, rather than being exposed to threats in Mozambique. The study area therefore forms a template that can inform management strategies and research priorities.

The seasonality effect is not expected to strongly predict elasmobranch communities in a subtropical zone like iSimangaliso, due to considerably homogeneous environmental conditions through time. In this study, it seemed most elasmobranchs, in particular rays, were residents in the area year-round, with two ray species and one small-bodied shark species showing seasonal variation. Large-bodied mobile sharks were rarely detected with no obvious environmental correlations. Therefore, sampling throughout both seasons is necessary to assess the full spectrum of the shark and ray assemblages. The association of some species with higher or lower SBT can be easily explained by the seasonality of their observations, given that temperature is a proxy for season. It is difficult to accurately determine if temperature or some other variables were driving the species seasonal patterns.

Most species were sighted in the entire depth range of this study (from 12 to 33 m depth), but whiprays seemed to be more common between shallow and intermediate depths (<25 m depth). Conversely, *C. humani* was more common in the deeper zone in this study. This species is known to reach 43 m deep (Pollom et al., 2019; White & Weigmann, 2014) and past research in iSimangaliso found this shark to be common deeper than 30 m, with the deepest record at 90 m depth (Bernard, personal observation, 28 March 2023). This study only sampled the shallow portion of the depth range for this species due to logistical constraints associated with the methodology. *Carcharhinus humani* showed a distinct seasonal pattern with individuals more common in shallower zones during summer. This pattern may reflect a seasonal depth change that warrants further investigation, potentially incorporating acoustic telemetry tracking.

In spite of continuous environmental variables only contributing to 7.35% of variation in elasmobranch composition in this study, environmental drivers may present influence on a species-specific scale. There may be unexplored factors which are stronger drivers for shark and ray assemblages and assessing other abiotic variables (*e.g.*, current direction) could provide further insights.

Location was not a significant driver of elasmobranch species composition. Nevertheless, submarine canyons can have a recognized impact on abiotic conditions (*e.g.*, upwelling of cooler deep waters) (Allen et al., 2001), and it is known that big pelagic sharks can be associated with these geomorphic features (Bradford et al., 2020; Klimley et al., 2002; Yano et al., 2007). Further research in the study area is necessary to assess the possible effect of the Diepgat canyon on the local elasmobranch communities.

### Applicability of the methodology

The number of identified elasmobranch species in this research was lower when compared to other stereo-BRUVs assessments in South Africa, which may be due to other studies having larger sampling sizes over a longer period, the inclusion of assessments of different habitats besides sand, the inclusion of greater depths, and a higher diversity of endemic species in the study areas (Cortelezzi et al., 2022; De Vos et al., 2015; Osgood, McCord & Baum, 2019). Thus, it would be of interest that future research near the sampling area compares iSimangaliso’s sand and reef habitats.

In this study, underwater visibility was not significantly different between seasons, which suggests water clarity is suitable enough year-round for monitoring using this methodology. Footages of stereo-BRUVs allowed for the visualisation of biological variables, such as size and sex, which can be assessed in further studies. Although the deployments allowed for the observation and counting of elasmobranch individuals, stationary equipment may underestimate true relative abundance and diversity when using the conservative measure MaxN (Kilfoil et al., 2017; Sherman et al., 2018). It should therefore not be unquestionably assumed that the species only sighted in a certain season or depth zone in this study are completely restricted to those features. Thus, longer-term monitoring studies using stereo-BRUVs will help create a stronger database to assess patterns in elasmobranch communities; for instance, recognizing interannual variability in the presence of large and highly mobile species.

## Conclusions

This study contributed to the understanding of the elasmobranch communities of a sandy habitat, as well as its relationship with season and the surrounding environmental conditions. In this ray-dominated study area, season and its proxy temperature, together with water depth were significant in shaping elasmobranch assemblage composition. However, almost all species were present in both seasons. This finding supports the concept of MPAs which provide year-round protection as opposed to time-area closures which, despite being beneficial during feeding/mating/pupping events, cannot ensure adequate continuous protection to elasmobranch species. Most species sighted during the surveys had at least a “Vulnerable” conservation status, and the “Critically Endangered” *R. djiddensis* was notably abundant. Thus, it is essential to ensure the enforcement of the MPA rules in this “Controlled” area, in order to achieve an effective preservation of all the sharks and rays that either reside in or pass through this region. The stereo-BRUVs methodology proved to be an efficient technique to survey elasmobranchs in a quick, non-invasive and repeatable manner, which is particularly important in seasonal studies and in the management of MPAs where resources are often constrained. As the first study in this area, further research is necessary to incorporate adjacent deeper habitats and assess long-term seasonal trends of the local elasmobranch communities.

##  Supplemental Information

10.7717/peerj.15636/supp-1 Supplemental Information 1 Environmental and biological data of each stereo-BRUVs sampling site Deployment details (including measurements of environmental variables), and the species MaxN associated with each sampling site, during winter and summer seasons.

## Additional Information and Declarations

### Field Study Permissions

The following information was supplied relating to field study approvals (i.e., approving body and any reference numbers): Field experiments were fully approved by the iSimangaliso Wetland Park Authority.

### Data Availability

The following information was supplied regarding data availability: The raw data are available in the Supplemental Files.
